# On the forest cover–water yield debate: from demand- to supply-side thinking

**DOI:** 10.1111/j.1365-2486.2011.02589.x

**Published:** 2012-03

**Authors:** David Ellison, Martyn N Futter, Kevin Bishop

**Affiliations:** * Institute for World Economics, Hungarian Academy of Sciences1014 HU Budapest, Hungary; † Department of Forest Ecology, Swedish University of Agricultural Sciences90183 SE Umeå, Sweden; ‡ Department of Social and Economic Geography, 90187 Umeå UniversityUmeå, Sweden; § Department of Aquatic Sciences and Assessment, Swedish University of Agricultural Sciences75007 SE Uppsala, Sweden; ¶ Department of Earth Sciences, Uppsala University75236 SE Uppsala, Sweden

**Keywords:** afforestation, climate change adaptation, forest ecosystem services, precipitation recycling, water yield

## Abstract

Several major articles from the past decade and beyond conclude the impact of reforestation or afforestation on water yield is negative: additional forest cover will reduce and removing forests will raise downstream water availability. A second group of authors argue the opposite: planting additional forests should raise downstream water availability and intensify the hydrologic cycle. Obtaining supporting evidence for this second group of authors has been more difficult due to the larger scales at which the positive effects of forests on the water cycle may be seen. We argue that forest cover is *inextricably* linked to precipitation. Forest-driven evapotranspiration removed from a particular catchment contributes to the availability of atmospheric moisture vapor and its cross-continental transport, raising the likelihood of precipitation events and increasing water yield, in particular in continental interiors more distant from oceans. Seasonal relationships heighten the importance of this phenomenon. We review the arguments from different scales and perspectives. This clarifies the generally beneficial relationship between forest cover and the intensity of the hydrologic cycle. While evidence supports both sides of the argument – trees can reduce runoff at the small catchment scale – at larger scales, trees are more clearly linked to increased precipitation and water availability. Progressive deforestation, land conversion from forest to agriculture and urbanization have potentially negative consequences for global precipitation, prompting us to think of forest ecosystems as *global public goods*. Policy-making attempts to measure product water footprints, estimate the value of ecosystem services, promote afforestation, develop drought mitigation strategies and otherwise manage land use must consider the linkage of forests to the supply of precipitation.

## Introduction

Water availability – both now and in the future – is of the utmost importance. However, the role of forests, their impact on precipitation, water yield and the hydrologic cycle more generally remain hotly contested. Afforestation strategies to ameliorate dry season flows have come under increasing scrutiny and attack (Calder, 2002; Jackson *et al*., 2005; Trabucco *et al*., 2008; Malmer *et al*., 2009). Although the global warming and climate change adaptation potential of forests and associated ecosystem services are mobilized to boost potential carbon sequestration, fossil fuel substitution and biodiversity protection; the potentially beneficial relationship between forest cover and water yield is strongly questioned, even pilloried (Greeff, 2010). Thus, the potential for forests to improve, protect and promote water yield may be underutilized.

The fundamental assertion that ‘trees *use* water’ is at the root of these discussions. Though in important ways this simple fact is true, even mundane, what are often taken to be its principal implications are anything but. Many on what we call the ‘*demand-side*’ of the forest cover***–***water yield debate see trees and forests as consumers of available water and competitors for other downstream water uses (agriculture, energy, industry, households). This view, however, misses the *beneficial* side of this consumption. The same evapotranspiration (ET) that consumes water at one scale supplies water to the atmosphere, facilitating its cross-continental transport and promoting precipitation at local, regional and global scales.

Thus, we divide the forest water debate into two schools of thought: the ‘*demand-side*’ and the ‘*supply-side*’ schools. The latter supports the beneficial impact of forest cover on the hydrologic cycle, emphasizing that increasing forest cover raises water yield. Though some attribute *debate* over the outcome of increased forest cover on water yield to the gap between public perception and scientific knowledge (Calder, 2002), the scientific community itself remains sharply divided.

Current policy efforts suggest a possible shift in the perception of the benefits of forest–water interactions – for example, the European Commission's recent draft on forest policy (European Commission, 2010) or international attempts to estimate the value of ecosystem services (TEEB, 2010). However, a brief perusal of other strategic documents presents a different picture. On the basis of a number of attempts to define policy related to the water footprint (Hoekstra *et al*., 2009), ‘virtual water’ (Allan, 1998; Merret *et al*., 2003), the Water Framework Directive (WFD, 2000/60/EC), the development of drought management strategies and discussion of the role of afforestation, we illustrate that scientific discord is the order of the day. Crafting adequate and timely responses to the threat of global warming, climate change and increasing demands on water resources, however, requires timely resolution of the forest cover–water yield debate.

In contrast to the conventional demand-side small catchment (<1 km^2^ to ca. 10 km^2^) literature, we argue forest cover increases water supply at regional and global scales, in particular through the intensification of the water cycle. Precipitation (*P*) recycling not only raises the likelihood of local *P* events, it also favors the cross-continental transport of moisture vapor and thus increased *P* in locations more distant from the ocean-based hydrologic cycle. In this way, the climate regulatory function of forests has a beneficial impact on the water regime and the availability of water resources.

The list of forest–water interactions with relevance for water management and atmospheric regulation extends well beyond this discussion to phenomena such as water quality/purity, infiltration and groundwater recharge, soil water storage, flood moderation/prevention and atmospheric cooling (Malmer *et al*., 2010; Ellison, 2010). Due to space limitations, we focus herein on the set of *intensifiers*: forest–water interactions that promote an increased rate of circulation in the hydrologic cycle. We aim first to highlight the demand- and supply-side debates and briefly sketch the problems raised by questions of scale. Second, we illustrate the potential importance of the supply-side by emphasizing the role of forests and other land uses for the production of moisture vapor and potential contributions to cloud formation and precipitation. Third, we highlight the continental, distributional and seasonal impacts of source contributions to precipitation and clarify how this model impacts and modifies conventional views of the hydrologic cycle. Fourth and finally, we discuss potential theoretical and policy implications.

## Understanding water supply, quantity and balance

One of the most poorly understood (though by no means under-researched) forest–water interactions is the impact of forests on water yield. The scientific literature is broadly divided between researchers working with very local microperspectives and researchers modeling at larger spatial scales.

The local, *demand-side school* findings suggest that reafforestation reduces available water supply. This is based on a number of small-scale studies. As tree cover increases terrestrial interception, evaporation and transpiration (hereafter referred to as evapotranspiration or ET), reafforestation reduces runoff, leading to declining water availability, particularly in areas not forested for long periods of time (see Hibbert, 1967; Bosch & Hewlett, 1982; Zhang *et al*., 2001; Calder, 2002; Andréassian, 2004; Brown *et al*., 2005; Farley *et al*., 2005; Jackson *et al*., 2005; Malmer *et al*., 2009). Clear-cutting and deforestation, on the other hand, lead to a period of increased water availability, at least until the forested state returns. In these empirical studies, the presence of forest vegetation typically removes water from the local hydrologic cycle, reducing local water supply.

In the *demand-side* view, forests compete against other important water-consuming activities (agriculture, energy, industry, households). Thus, the ecosystem services forests provide (e.g., biomass production) are included on the demand-side of the water balance equation. Climate change raises the stakes with regard to future water availability. As trees *use water*, considerable care should be given to how and where trees are planted. Afforestation strategies in particular have come under increasing scrutiny (Calder, 2002; Jackson *et al*., 2005; Trabucco *et al*., 2008; Malmer *et al*., 2009). Greeff (2010) provides a particularly impassioned plea for restraint in the planting of forest plantations in more arid African zones.

Tree type can also impact the local water balance. Eucalyptus trees in particular have been criticized in the more arid regions of Africa for their heavy drain on available water supply (RELMA, 2003; SciDev.Net, 2009). Deciduous tree species typically generate less ET than evergreen, potentially mediating forest impact on the local water balance. Wattenbach *et al*. (2007) find evidence that planting deciduous species such as oak in temperate zones diminishes negative effects on the water balance resulting from the increased ET associated with evergreen species. Overall, however, regardless of the tree species, the effect on the water balance is typically assumed to be negative (see also Bosch & Hewlett, 1982; Jackson *et al*., 2005; van der Salm *et al*., 2006; Rosenqvist *et al*., 2010).

The *supply-side school* on the other hand suggests that the overall impact of forests is one of improving water availability at the regional and/or global scale. Sheil & Murdiyarso (2009) and the work they draw upon most heavily (Makarieva *et al*., 2006; Makarieva & Gorshkov, 2007; see also Savenije, 1995; Gordon *et al*., 2005; Kravčík *et al*., 2008) represent the most profound departure from the *demand-side* school, arguing that large forest expanses should be seen as ‘biotic pumps’, drawing in moisture from the earth's oceans and driving continental ET and *P*, thereby replenishing, renewing and intensifying the regional water cycle. Without large forest expanses, the intensity of the water cycle diminishes, reducing water availability.

Millán *et al*. (2005) and Millán (2008) arrive at similar conclusions for the regional water cycle in the coastal zones of the Mediterranean. They argue that deforestation has two important consequences. First, moisture coming in from the sea, typically in the form of fog, is no longer trapped along the coast. Second, increased land temperatures due to vegetation loss result in rising vertical circulation columns that carry storms up and over surrounding mountains, reducing orographic precipitation. Like Makarieva *et al*. (2006) and Makarieva & Gorshkov (2007), though based on different mechanisms, Millán *et al*. (2005) and Millán (2008) argue that vegetation loss leads to lower ET and subsequently lower *P*. Produced ET is carried away to other places and no longer deposited locally as *P*.

Regionally, deforestation has been linked to reduced *P*, increased low flow events, extended dry spells and drought. Costa & Pires (2009), for example, find that deforestation in the Amazon and Cerrado regions of Brazil resulted in average increases in the dry season of one full month. Wang *et al*. (2009) present similar findings for a sub-basin of the Yellow River in China. Osborne *et al*. (2004) find a significant relationship between increased forest vegetation cover, increased *P* and reduced surface temperatures using global modeling techniques. Reviews of 26 studies in the Amazon (Marengo, 2006, p. 11) and 14 deforestation studies (Hahmann & Dickinson, 1997) find support for the connection between deforestation and declining *P*.

Other recent literature points to more ambiguous findings. D'Almeida *et al*. (2007) note, for example, that while a large number of large-scale modeling predictions suggest deforestation leads to declining *P* and reduced runoff (*R*), many local-scale observations find reduced ET and increased runoff. Meso-scale observations are more ambiguous. And Silva Dias *et al*. (2009) suggest that the atmospheric dynamics unleashed by deforestation can lead to increased *P* in some areas. A strict accounting budget would require however that, even with increases in *P* in some locations, total regional *P* must decline. Silva Dias *et al*. (2009) themselves point out that about half of the moisture required for *P* in the Amazonian region is supplied by the rainforest. Thus, reductions in ET should mean overall reductions in *P*. Garcia-Carreras & Parker (2011) may provide the beginnings of an explanation for this anomalous finding. In a study of a West African region, the authors found that *P* increases over deforested areas as a result of fishbone-style deforestation. This came, however, at the expense of *P* reductions of 50% or more over forested areas. Relaxing their modeling assumption of constant total moisture, the impact of reduced ET from deforestation and reduced rainfall over forests should imply reduced total *P*.

## A question of scales

Few authors bridge the divide between local and regional scales in the forest–water interaction literature. Important weaknesses regarding spatial and temporal scales emerge in both literatures. The methodological problem of scale appears to be a major issue separating these two camps. Andréassian (2004, pp. 9 and 18) notes that watershed size in the vast majority of paired-catchment studies is less than 2 km^2^, which is understandable as such studies cannot be conducted on much larger catchments. Catchment basins of this size can easily receive precipitation from other locations and the impacts of change in a catchment (e.g., deforestation, reduced ET and declining *P*) may become evident only further away.

On the other hand, both Makarieva *et al*. (2009) and Sheil & Murdiyarso (2009) argue that broad expanses of forest (presumably significantly greater than 2 km^2^) give rise to increased *P*. Continuous forest cover from the sea moving inland facilitates water vapor transport further inland. Measured at smaller scales, the potential impact of increased forest cover on water availability and *P* may not be present, may not be easily observable or may escape statistical significance. Thus the demand- and supply-side schools – because they address fundamentally different scales – may simply measure and talk past each other.

Timescales of ecosystem regeneration represent a further complication. Most studies of the impact of reafforestation consider relatively short periods of 1–10 years (Brown *et al*., 2005). However, time presumably matters in the forest–water relationship. As other authors have concluded, ecosystem regeneration can take long periods of time (cf. Parotta *et al*., 1997). Soil properties may take the longest to recover. Diochon *et al*. (2009) find that postharvest soil carbon storage first reaches a *minimum* after 32 years of growth – at a level only 50% of the soil carbon storage in intact forests – and reaches 100% only after 100 years. Forest ecosystems potential for soil water storage and groundwater recharge presumably also require exceptionally long regeneration periods.

Findings in the deforestation literature contrast sharply with those of the *demand-side school* and raise significant questions. Paired-catchment studies provide evidence for a positive relationship between deforestation and local water availability. At larger scales, deforestation studies suggest increasing levels of forest cover should raise *P* levels and river runoff. Such contradictions, however, are rarely addressed and no clear explanation emerges for why deforestation studies typically find declining water availability. Nor is there much discussion about the inverse proposition that increases in forest cover may lead to increasing water availability. Furthermore, change in water supply may be limited by such factors as diminished soil quality and quantity (soil carried away by erosion cannot be quickly replaced). Limited empirical evidence is available however to substantiate or reject these claims.

## From demand- to supply-side thinking: on recycling rainfall

### From forest ecosystems to ET

By convention, whether at the global or local scale, accounting for total available water supply focuses almost entirely on the *demand-side* of the equation. In its simplest form, total available water is given by *P*, which is apportioned into ET and *R*. Change in storage (*ΔS*) is considered negligible, so any increases in ET necessitate a decrease in *R*.

**Fig 1:**
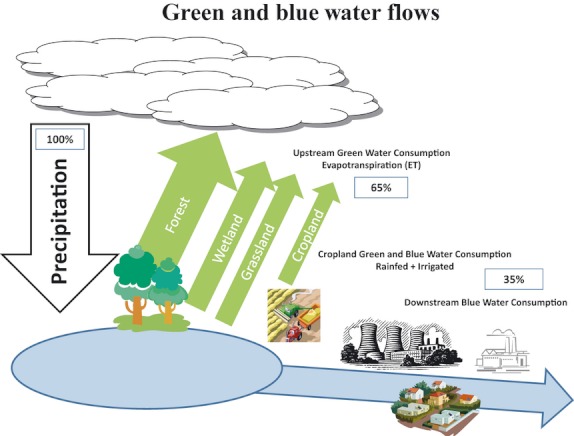
Green and blue water flows adapted from Falkenmark (2009).

Dividing the water balance into *green* and *blue* water flows further accentuates the demand-side view of trees by distinguishing *blue* waters available for consumption purposes from the losses to blue water deriving from *green* water flows or transpiration, that part of ET contributing to plant growth. *Green* water flows are primarily attributed to vegetation growth, in particular forests and agriculture. The sum of *green* and *blue* water flows defines the water balance and *blue water* (*R*) is the total amount of water available for aquifer recharge and consumption by irrigated agriculture, the energy sector, industry, households and others. As *green water* (ET) is transpired by plants, it is not available for consumption and thus is counted as reducing available *blue water* (Fig. 1). Although *green water* may include *productive* consumption by such sources as rain-fed agriculture, the primary consumer and thus source of *green water* is natural vegetation. Rockström & Gordon (2001), Rockström *et al*. (2009) and Hoff *et al*. (2010) thus argue that green water flows – the *productive* consumption of available water supply – must be considered on the *demand-side* of the global water balance (see also Falkenmark, 2008; Gleick & Palaniappan, 2010).

With the notable exception of Gordon *et al*. (2005), few authors on the *demand-side* consider the *supply-side* of the water balance equation. Yet ET should presumably be counted as an *addition to* the global water balance. In this sense, the real ecosystem service of vegetation, in particular forests and woodlands, may be their ability to return moisture vapor to the atmosphere and thus intensify the water cycle. In fact, this ecosystem service is responsible for as much as one-third of total global ET (Rockström & Gordon, 2001, p. 847). In this regard, Makarieva *et al*. (2006, p. 898) argue against ‘setting *P* as an independent variable in the water cycle problem’. Maes *et al*. (2009) likewise view ET as a *contribution to* rather than as an *extraction from* the overall water balance.

In this sense, trees are literally *recyclers* that draw upon available water supply and pump it back into the atmosphere. Trees not only *attract* rain (cf. Andréassian, 2004), but they also can and do recirculate moisture back into the atmosphere in the form of ET. Moreover, the rate at which moisture is recirculated in the form of ET will in turn have an impact on the frequency and even potential duration of *P* events. Thus, rather than the traditional water balance formula given by *P* = ET + *R* (river runoff), we should be thinking about the supply-side, where: ET + OE = *P* (Makarieva & Gorshkov, 2007: 898). In particular because ET can be strongly influenced by a broad range of land use choices, we adopt this form of the equation, separating OE (total evaporation from oceanic bodies) from terrestrial ET. Thus the greater (smaller) is ET, the greater (smaller) is *P*. Given that the magnitude of OE is not easily manipulated, in what follows we focus our attention on the sources of ET.

The concept of green and blue water flows can be turned on its head and used to estimate the value of ecosystems in supplying ET and thus *P*. Using data from Rockström & Gordon (2001), we estimate the relative importance of various forms of land cover in their contribution to ET. In particular, we focus on the relative evaporative efficiency of different types of vegetation cover (Table 1a), and their potential contribution to the global *P* budget (Table 1b).

**Table 1a:** Evaporative potential: global green and blue water sources

	Area (10^6^ km^2^)	ET (10^3^ km^3^ year^−1^)	Evaporative potential (m y^−1^)
Rockström & Gordon (2001)			
Forest and woodlands	55.46	40	0.72
Wetland	1.704	1.4	0.82
Grassland	29.54	15.1	0.51
Cropland	17.6	6.7	0.38
Oki & Kanae (2006)			
Forest	40.1	29	0.72
Wetland	0.2	0.2	1.00
Grassland	48.9	21	0.43
Cropland	15.6	7.6	0.49
Other	26.4	6.4	0.24
Evaporation from water body surfaces			
Lakes	2.7	1.3	0.48
Oceans (Trenb.)	362	413	1.14
Oceans (O&K)	362	436.5	1.21

**Table 1b:** The ET-multiplier

	Rockstrom & Gordon (2001)	Hubbart & Pidwirny (2010)	Oki & Kanae (2006)	Trenberth *et al.* (2007)
ET-Multiplier				
Share of ET in *P*	61.2%	63.9%	59.0%	64.6%
Ratio Terrestrial ET to oceanic evaporation	n.a.	1.78	1.44	1.83
Ratio terrestrial ET to blue water flows	1.58	1.78	1.44	1.83

ET, evapotranspiration. All data on global evaporative potential and the ET-multiplier has been calculated based on available data from the following sources. Evaporative potential is estimated based on primary data from both Rockstro_m & Gordon (2001) and Oki & Kanae (2006). The Rockstro_m & Gordon (2001) data required some substitution. Total cropland area is from Matthews (1983). Evaporative efficiency from water body surfaces has been calculated based on data from Oki & Kanae (2006) for lakes and oceans and from Trenberth et al. (2007) for oceans. Hubbart & Pidwirny (2010) provide an oceanic evaporation estimate that is approximately the average of the other two sources. Ocean area was obtained from Eakins & Sharman (2010). Calculations of the ET-Multiplier in Table 2 are based on data from multiple sources as indicated in the table.

ET (and thus the ET-multiplier) is clearly affected by the relative evaporative efficiency of different kinds of vegetation or land cover. Considering the *evaporative potential* of a unit of land with different kinds of vegetation cover (calculated in Table 1), forest and wetland cover is almost twice as efficient as other forms of vegetation cover. This finding is supported by Farley *et al*. (2005, p. 1571), who attribute this effect to both the larger leaf area index (LAI) of trees and their ability to access deeper water resources as a result of greater root depth. Kleidon & Heimann (2000) likewise point to the relative importance of root depth in promoting ET. Even inland water body surfaces exhibit lower rates of evaporative potential. By convention, *sealed surfaces* (urban land) are typically considered to have no ET potential. Although not strictly true, sealed surfaces can and do give rise to some ET (Maes *et al*., 2009, p. 7326), we likewise adopt this simplifying assumption. Thus, both land conversion to agriculture and expansion of the urban environment have important impacts on total available ET and thus *P*.

The share of ET in total *P* ranges from 59% to 64% (Table 2). More importantly for our purposes, the ratio of green to blue water flows – what we call the *ET-multiplier* – is high, ranging from about 1.6 to 1.8. It is also relatively stable across different source estimates. This number is equivalent to the ratio of green water to the oceanic contribution to precipitation. The *ET-multiplier* measures the relative proportion of moisture vapor terrestrial vegetation can return to the atmosphere. The more vegetation is available for turning rainfall into moisture vapor, the higher the ET-multiplier and the more moisture vapor will be available for generating new precipitation. As a ratio, the ET-multiplier represents the relative importance of the *terrestrial ET contribution* relative to the oceanic contribution.

**Table 2:** Average contribution and ratio of local and total terrestrial precipitation sources averaged across all basins

	Local (%)	Terrestrial (%)	Ratio (*T*/*L*)
Annual	13	40	3.0
DJF	10	29	3.0
MAM	15	43	2.9
JJA	16	48	3.0
SON	13	40	3.0

Own calculations based on Bosilovich data. ET, evapotranspiration; DJF, winter; MAM, spring; JJA, summer; SON, fall.

Given the importance of ET for generating regional *P*, current rates of deforestation are a significant cause for concern and are likely to lead to declining water availability. According to Hansen *et al*. (2010), in only a few years from 2000 to 2005 the world lost approximately 3% of global forest cover. Assuming forest cover loss is globally evenly distributed, the global ET-multiplier would have been reduced to approximately 1.56 (from 1.58). However, where deforestation is more concentrated, we can expect greater impacts on the ET-multiplier. Historically, of course, these magnitudes have been much greater. Global wetlands are thought to have declined by some 50% during the 20th century (Revenga *et al*., 2000, pp. 3, 21–2), further reducing ET. Land use changes thus have potentially significant implications for *P* loss further across continents.

Land conversion type affects the ET-multiplier. Although converting forests to urban environment produces the greatest reduction in ET impact, *P* loss should be only half the size if forests are converted to agriculture. However, this still represents a potentially significant loss to the water balance. Moreover, land conversions are common. For the period 2000–2005, DeFries *et al*. (2010) find the principal explanatory factors of tropical deforestation were urbanization and agricultural exports. Gibbs *et al*. (2010) find that between 1980 and 2000, some 83% of new agricultural land in the tropics resulted from forest land conversion. Wetland loss is commonly attributed to similar causes.

As all ET flows ultimately return as *P* (very small amounts of moisture vapor can escape the outer atmosphere), the principal conclusion from the aforesaid findings is that *all vegetation-based production of ET* should be thought of as an *ecosystem service*, in particular forest- and wetland-based ET. Given the higher ET coefficient of vegetation, especially wetlands and tall vegetation like forests, their removal will significantly reduce ET, leaving the earth's surface with lower *P* and more limited *R*. Thus, at some larger scale, the total amount of regional and global precipitation is directly linked to ecosystems and global ET.

As trees *use water*, afforestation effects on the local water balance may be significant, particularly in arid or semi-arid regions. The other side of the equation, however, must also be considered. In particular where ecosystems are not significantly or severely water-constrained, additional forest area can ostensibly be tolerated and can provide an important contribution to the global water balance and to local areas where water is in particularly short supply.

Thus, the primacy of ET and the ecosystems that provide it should presumably be set equal to or even supersede *demand-side* representations of forests as consumers of available water supply. In this ‘revised’ view, one of the principal roles of forest ecosystems is precisely their ability to produce ET. Overemphasis of the fact that *trees use water* fails to provide an adequate framework for understanding one of the principal values of forest ecosystems. These are defined not only by the ‘end-products’ they produce (biomass, carbon sequestration, biodiversity, etc.), but also by the centrality of their role in atmospheric regulation and the production of ET.

### From supply (ET) to *P*

ET is not lost to the system but is returned as *P*. Once moisture is returned to the atmosphere as ET, the critical question for forestry's impact on water availability at larger geographical scales is where that moisture falls. If that moisture ends up on land, then there is a strong case for forests intensifying the continental component of the hydrologic cycle. Next, we highlight the set of *intensifiers*: forest–water interactions that ‘attract’ or *promote* rain on land surfaces, thereby promoting an increased rate of circulation in the terrestrial hydrologic cycle.

Higher relative humidity, a function of both temperature and the amount of water vapor, will raise the likelihood of *P*. A 10% rise in humidity can lead to 2–3 times the amount of *P* (Fan *et al*., 2007, p. 14; Khain, 2009, p. 12). By converting heat to energy, forests also generate cooler temperatures, increasing the relative humidity produced by a given amount of water vapor and contributing to potential *P*. Aerosols emitted by trees further promote condensation and thus cloud formation (Fan *et al*., 2007; Khain, 2009; Pöschl *et al*., 2010). Finally, positive interactions between aerosols and relative humidity likewise promote higher levels of precipitation (Fan *et al*., 2007).

Forests also have the ability to passively ‘attract’ or ‘catch’ atmospheric moisture, in particular humidity in fog and clouds. Condensation on plant surfaces provides additional moisture for tree growth (Bruijnzeel 2001, 2004; Millán *et al*., 2005; Pepin *et al*., 2010). Estimates suggest that anywhere from 200 mm year^−1^ to 425 mm year^−1^ additional moisture can be obtained through fog condensation (Azevedo & Morgan, 1974; del Val *et al*., 2006). Some also find a positive correlation between the surface roughness of forests (varied age class) and *drag* coefficients, a precursor for potential precipitation (Osborne *et al*., 2004; Hahmann & Dickinson, 1997). Equally important, a share of the moisture captured from the atmosphere in this way is again returned to the atmosphere as ET and remains available for vegetation growth in other areas (see, in particular, Pepin *et al*., 2010).

As forests and wetlands circulate greater amounts of moisture as ET, they contribute more to the relative *intensity* of atmospheric moisture vapor circulation than other land cover types. Increasing forest cover and density is therefore positively related to the potential for higher relative humidity and potential *P*. Although other variables such as temperature, wind speed and aspect also play a role (on sources and triggers, see Trenberth *et al*., 2003), the first two variables (total absolute amounts of vapor and heat) are positively and strongly correlated with the occurrence of *P*. On the other hand, questions remain about the relative complexity of these relationships, in particular the degree to which precipitation recycling is driven by questions of scale, relative density or critical mass (of forest mass, vapor mass, prevailing *T* and wind conditions), as well as factors such as slope, aspect, topography, wind and extreme heat (see, e.g., Silva Dias *et al*., 2009; Bisselink & Dolman, 2009; Gangoiti *et al*., 2011a,b).

The amount of recycled *P* is strongly dependent on the land area considered, with larger geographical expanses having greater potential for recycling. In large basins such as the Amazon, water recycling may be an important part of total *P*. Positive feedbacks between ET and *P* may also have significant effects on land cover. Thus, it has been argued that rainforests exist because moisture evaporated and transpired by trees is reprecipitated on an almost daily cycle (Salati *et al*., 1983).

Finally, forests likewise appear to influence atmospheric dynamics in important ways that both result from the production of ET and influence its transport. Thus some argue that the production of ET may itself induce changes in atmospheric pressure dynamics, driving the transport of water vapor across continental space. Transects inland from the coast through forests typically have much higher precipitation than those on nonforested lands (Makarieva *et al*., 2006; Makarieva & Gorshkov, 2007; Sheil & Murdiyarso, 2009; see also Savenije, 1995). Meesters *et al*. (2009), however, have suggested there are fundamental problems with the physics in the biotic pump hypothesis. Millán *et al*. (2005) and Millán (2008) also argue that the loss of forest cover operates in the reverse sense: relative deforestation raises land temperatures resulting in the formation of vertical circulation columns that carry storms up and over surrounding mountains, reducing orographic precipitation. Both authors agree that increasing forest cover positively influences atmospheric dynamics in ways that raise the likelihood of *P* events.

## Seasonality and the relative weight of oceanic, terrestrial and local sources of precipitation

The relative *intensity* or *rate* of precipitation and thus water cycling across different bioclimatic envelopes and forest biomes (Mediterranean, temperate, boreal, etc.) and its relationship to questions of forest mass, density, temperature and/or prevailing wind patterns remains uncertain. For the Amazon region, Marengo (2006) reviews and summarizes previous estimates, with local *P* recycling ranging from 38% to 82%. *Local* typically appears to be defined as recycling from the same river or catchment basin up to an area of a few thousand square kilometers. However, early attempts to estimate recycled ET simply defined it as the observed difference between *P* and *R*; recycled ET = *P* − *R*. Thus, as Marengo also suggests, much of the early literature on *P* recycling presumably greatly overestimates the actual degree of *local* recycling. Applying substantially different measures than those cited in Marengo (2006), Bosilovich & Chern (2006) and Bosilovich & Schubert (2002) find significantly lower levels of *local P*-recycling. But they also find that, at continental scales, the *terrestrial* share of *P* recycling is significantly higher than the *local* share (here defined as the river basin contribution). Moreover, though ‘local’ *P* recycling occurs, much of the ET will travel significantly greater distances before falling as *P* (Eltahir & Bras, 1994, 1996). Thus, just how *local* water recycling really is and how important this question is in the larger theoretical and empirical context remains unclear.

Thus whether *local*, *regional* or *terrestrial/continental* forms of *P* recycling should be the central focus remains an open scientific question. Most previous work has tended to focus on the *local* component of *P* recycling, ignoring contributions from terrestrial and oceanic sources. What appears to matter, however, in understanding the relative contribution of ET to *P* is the larger-scale contribution from *terrestrial* sources (alternatively Dirmeyer & Brubaker, 2007; Dirmeyer *et al*., 2009 focus on local contributions; see also van der Ent *et al*., 2010). Seasonality too is largely ignored, despite the fact that more ET is produced during the growing season, ‘intensifying’ the hydrologic cycle when it is most needed (see, however, Dirmeyer & Brubaker, 2007).

**Fig 2:**
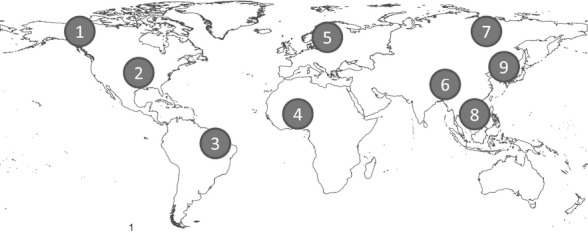
Major river basins and large-scale study sites used in regional recycling and precipitation source assessment. 1) Mackenzie river basin, 2) Mississippi river basin, 3) Amazon river basin, 4) West Africa, 5) Baltics, 6) Tibet, 7) Siberia, 8) GAME (GEWEX Asian Monsoon Experiment) and 9) Huaihe river basin.

To provide an estimate of the contributions from *terrestrial* and other sources, we use source data provided by Michael Bosilovich (for more detailed discussions of these data, see Bosilovich & Schubert, 2002; Bosilovich & Chern, 2006). Based on these data, we calculate the total *terrestrial*, *local*, *oceanic* and *polar* source contributions to *P* from the more disaggregated regional source contributions described in Bosilovich *et al*. (2002). We also calculate the projected seasonal contributions [winter (DJF), spring (MAM), summer (JJA) and fall (SON)] in the major river basins and large-scale study sites from monthly aggregated data. These data are derived from global climate models and generate predictions of the contribution of moisture vapor from different geographic locations to *P* in different river basins and large-scale study sites distributed around the globe (see Fig. 2). The simulations are run over a 50-year time period from 1948 to 1997 with these data averaged over the entire period. The methodology is described in Bosilovich & Schubert (2002) and more detailed information on the river basins is available in Bosilovich *et al*. (2002). Attempts to validate these predictions suggest bias estimates of approximately 0.2 mm day^−1^ (or ca. 5%) at the local scale. Estimates are more precise at the global scale (within ∼2%; Bosilovich & Schubert, 2002, pp. 153 and 156). The principal advantage of this dataset is its potential ability to track moisture vapor from global sources on a relatively detailed geographic scale.

Based on the results of our calculations from the Bosilovich data averaged across all basins, the average annual local contribution represents only about 13% of total precipitation (Table 3). However, at the larger regional/continental scale, the terrestrial source contribution is three times higher, representing approximately 40% of precipitation. At both the local and regional/continental scales, ET is a more important source of precipitation during the summer months (JJA), reaching as much as 16% locally and 48% of the terrestrial contribution to precipitation. Even in this case, however, the local component remains about one-third the size of the of the larger-scale continental or terrestrial component of precipitation. There is, however, considerable regional and seasonal variation across individual river basins and study sites. Estimated ratios of the continental/regional to local source components range from approximately 1.4 in GEWEX Asian monsoon experiment (GAME) to 6.5 in Siberia (Table 3). While oceanic sources can also be important, the relative oceanic share is likewise highly seasonal and varies greatly from region to region and river basin to river basin and study site.

**Table 3:** Average precipitation in% and mm per season in major river basins by source (1948–1997)

		Seasonal shares			Annual shares		Ratios	
River basins and study sites	Season	Local (%)	Terrestrial (%)	Oceanic (%)	Terrestrial (%)	Oceanic (%)	Terrestrial to local	ET-Multiplier
MacKenzie	DJF	4	11	88	37	60	2.8	0.1
	MAM	14	38	60			2.7	0.6
	JJA	18	**59**	38			3.2	**1.5**
	SON	8	25	71			3.3	0.4
Siberia	DJF	6	38	43	68	23	6.4	0.9
MAM		15	**72**	20			4.7	**3.7**
	JJA	14	**78**	18			5.5	**4.3**
	SON	9	**60**	26			6.5	**2.4**
Baltics	DJF	4	13	79	30	61	3.5	0.2
	MAM	10	36	54			3.6	0.7
	JJA	13	**51**	41			3.9	**1.2**
	SON	5	21	67			4.0	0.3
Mississippi	DJF	9	47	52	58	41	5.1	0.9
	MAM	19	**62**	38			3.3	**1.6**
	JJA	24	**65**	35			2.7	**1.9**
	SON	11	**51**	48			4.7	**1.1**
Huaihe	DJF	6	34	65	42	57	5.2	0.5
	MAM	7	42	57			6.2	0.7
	JJA	11	43	56			4.0	0.8
	SON	10	45	54			4.4	0.8
Tibet	DJF	16	30	68	46	52	1.9	0.4
	MAM	23	40	58			1.8	0.7
	JJA	27	**50**	48			1.8	**1.0**
	SON	28	**51**	48			1.8	**1.1**
GAME	DJF	5	10	90	22	78	2.0	0.1
	MAM	15	21	79			1.4	0.3
	JJA	16	25	74			1.6	0.3
	SON	12	19	80			1.6	0.2
West Africa	DJF	8	32	60	41	52	4.0	0.5
	MAM	10	42	53			4.2	0.8
	JJA	7	38	55			5.8	0.7
	SON	8	44	48			5.7	0.9
Amazon	DJF	28	43	55	40	59	1.6	0.8
	MAM	22	37	62			1.7	0.6
	JJA	14	24	76			1.8	0.3
	SON	29	44	55			1.5	0.8

Own calculations based on source data provided by Michael Bosilovich (NASA). Oceanic, terrestrial, local and polar moisture vapor sources are compiled from the more highly disaggregated sources available in Bosilovich's data. The local source is included in the terrestrial source. Polar sources include both the polar oceanic regions and the Mediterranean. Seasonal contributions have also been calculated based on monthly source data. ET, evapotranspiration; GAME, GEWEX Asian monsoon experiment; DJF, winter; MAM, spring; JJA, summer; SON, fall. The seasons are reversed in the Southern Hemisphere (Amazon river basin).

**Fig 3:**
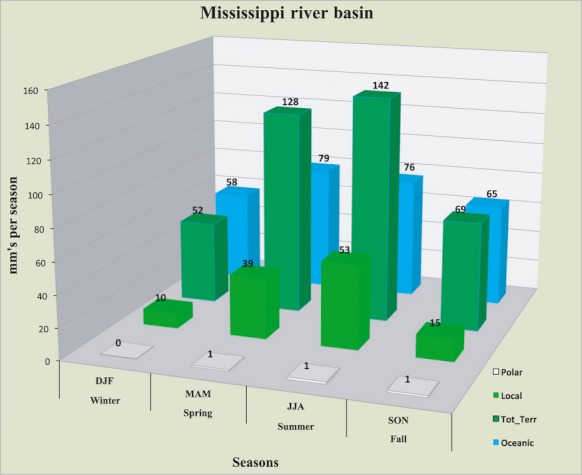
Average estimated precipitation in % and mm per season in the Mississippi river basin (1948–1997).

With the Mississippi River Basin depicted by way of example in Fig. 3, the data presented in Table 3 indicate strong seasonality in *P* recycling, with the continental and local contributions to terrestrial *P* peaking during summer months. Summer ET is thus one of the most important contributions to *P* in the world's major river basins and study sites. In over half the analyzed river basins and study sites, the terrestrial contribution to summer *P* represents the single largest *P* source, greater even than oceanic sources (occurrences of the terrestrial contribution are highlighted in bold, Table 3). In river basins and study sites relatively distant from coastal zones, in particular in Siberia (68%) and the Mississippi river basin (58%), summer terrestrial ET far outweighs other *P* sources (local or oceanic). This point highlights and underlines the importance of cross-continental moisture transport. For these basins, the ET-multiplier effect is comparatively high. Relative to the average ET-multiplier across all basins, study sites and time periods (0.7), for these basins the ET-multiplier is 2.8 and 1.4, respectively. However, even in river basins and study sites comparatively close to coastal zones, terrestrial sources can substantially contribute to local P. This is particularly pronounced in river basins and study sites at high latitudes. In the Baltics, the terrestrial contribution is about 30%. As noted before, the specifically *local* component of ET typically represents a far smaller share of total precipitation. However, during the summer months the local component can still represent a significant source, ranging from about 7% of total *P* in the West Africa study site to 28% in the Amazon basin.

Finally, although *P* recycling is sometimes associated with the Amazon river basin and a few isolated cases (such as cloud fog capture), these data suggest that *P* recycling is in fact a widespread phenomenon occurring across all the major river basins and study sites analyzed. For the Amazon, as with other river basins and study sites closer to the equator, the oceanic contribution is typically higher than other precipitation sources, in particular during the summer months (ranging from 55% in the Amazon, to 74% in GAME). On the other hand, for these regions, the specifically *local* contribution to *P* is frequently much higher relative to the continental contribution. This is true for the Amazon, GAME and surprisingly Tibet, but not for the Huaihe basin or West Africa. Thus, for the Amazon, the ratio of the terrestrial to the local contribution is only 1.6 (Table 3), much lower than the average of 3 across all basins and seasons (Table 3).

van der Ent *et al*. (2010) arrive at broadly similar findings. Their data suggest that the amount of precipitation recycling increases with the spatial scale considered. They estimate continental precipitation recycling ratios of between 1.25 for Oceania and 1.95 for Africa. Overall, they estimate an average continental precipitation recycling ratio of 1.67, suggesting that ‘there is at least 67% more precipitation on the continents than in the hypothetical case where there is no continental feedback at all’ (van der Ent *et al*., 2010).

Several important implications emerge. Deforestation in all areas of the world will lead to smaller amounts of recycled *P* and thus reduced amounts of total *P* at continental scales. This is particularly problematic in the context of global warming and climate change. In the more arid regions of the world, this is also likely to mean a higher likelihood of drought as well as an expansion of the total drought-prone area. By the same token, *reafforestation* in Europe or in other parts of the world – in particular outside the tropics – can increase *P* and the overall intensity of the hydrologic cycle, especially during warmer periods of the year and potentially in a warmer future climate.

The ability of regions more distant from OE to withstand the more arid summer months depends almost entirely upon the ET potential of the remaining upwind part of the continent. Where extensive deforestation has taken place in upwind areas, one can expect increasingly warm and arid summers to have an increasingly negative impact on regional sustainability and adaptation potential. One can further expect the increasing likelihood of drought events with significant forest and other vegetation dieoff in the more downwind regions. These phenomena will most strongly influence interior and downwind continental regions. Moreover, this suggests the potential for important feedback effects resulting from the combination of deforestation and global warming. In this sense, forest cover represents an important sustainability component for other water uses, vegetation types and even agricultural production due to its potential to return large amounts of water back to the atmosphere. Thus, reafforestation efforts should presumably have important adaptation-related impacts on the overall hydrologic cycle.

Finally, land conversions, in particular from forest to other types of land use should be seen in a different and broader context than has currently been the case. The specifically *transboundary* nature of ET means that reduced forest cover in one area leads to reduced *P* in another. Thus, many land conversions appropriate water resources from other regions (Dirmeyer *et al*., 2009).

## Popular (mis)representations of the hydrologic cycle

Standard representations of the hydrologic cycle entirely fail to capture these dynamics. Though an iconic part of our understanding of how water is cycled, traditional *textbook* representations perpetuate the *demand-side* view of the hydrologic cycle and downplay or even ignore the role of forests, ET and cross-continental transport (for state of the art representations, see Oki & Kanae, 2006; Trenberth *et al*., 2007; Hubbart & Pidwirny, 2010). Based on our cursory analysis of some 40 generic representations, the literature typically focuses either on *oceanic* or *terrestrial* cycles. Few of these representations attempt to combine these two cycles. Most representations suggest that *oceanic* or *terrestrial* cycles are closed, self-contained systems. Oceanic cycles are typically illustrated with evaporation from the ocean surface transported over land and deposited as *P*. This P then returns to the ocean as river runoff or is emitted back into the atmosphere as ET.

Attempts are rarely made to consider the pivotal importance of forest cover and ET in cross-continental moisture vapor transport and subsequent terrestrial *P*. Moreover, almost no representations deal adequately with the ratio of terrestrial to oceanic contributions to terrestrial *P*. As the vast majority of OE falls back over the ocean as *P* (ca. 90%; Oki & Kanae, 2006; Trenberth *et al*., 2007), the ratio of ET to the oceanic contribution to terrestrial *P* is quite large, ranging anywhere from 1.4 to 1.8 times the oceanic contribution (Table 2).

As Dirmeyer & Brubaker (2007) emphasize:

All freshwater on or beneath the land surface arrived as precipitation, and ultimately all of that water was evaporated from the oceans. However, it may have taken multiple ‘cycles’ of precipitation and evaporation for any single water molecule to work its way from the ocean to a given terrestrial location, with evaporation from the land surface or transpiration through the terrestrial biosphere occurring in the intermediate cycles (p. 20).

Thus, despite the importance of ET to terrestrial *P*, its contribution is routinely under-represented in popular illustrations of the hydrologic cycle.

## Forest–water interactions and policy-making

These findings should help restructure our thinking on some of the most basic aspects of modern hydrology theory. Thus for example, where authors make claims like, ‘Freshwater resources are fundamental for maintaining human health, agricultural production, economic activity as well as *critical ecosystem functions*’ (Gleick & Palaniappan, 2010, our emphasis), this sentence could be rewritten with forest ecosystems and ecosystem functions at the center: ‘*Forest ecosystems and ecosystem functions* are fundamental for providing and maintaining freshwater resources, maintaining human health, agricultural production and economic activity’. Without forest ecosystems, freshwater resources would be reduced or eliminated over large expanses of the terrestrial landscape. Thus, forest ecosystems should be placed at the center of models related to water supply and thus human welfare. Rather than focusing primarily (or even exclusively) on *demand-side* relationships (consumption) it is *at least* equally important to consider *supply-side* relationships. Without this, one cannot understand either some of the principal factors driving the limitation of water supply (deforestation) or factors that may contribute to increases in supply (reafforestation).

As the terrestrial landscape is altered by increasing urbanization and agricultural production, the potential for continuous intensive precipitation recycling and cross-continental transport will decline. Given current and historical rates of deforestation, the earth is presumably massively below potential/historical ET and thus precipitation values. Although Europe and North America have exhibited increasing forest cover during much of the 20th century, historical estimates of past forest cover suggest extensive deforestation as a result of population growth and agricultural production (Kaplan *et al*., 2009). Global loss of forest cover and wetlands has greatly reduced the potential ET-multiplier. Thus, to rephrase a recent statement from the Copenhagen Climate Council statement ‘*if mitigation is about energy, then adaptation is about **[forests and]** water*’ (Clausen & Bjerg, 2010, p. 5; our addition and emphasis). Although convention assumes that the earth's water resources are finite (see, e.g., the Economist, 2010), our analysis suggests something different: the global hydrologic cycle is dependent on the atmospheric regulation provided by forests, in particular on the relative rate and intensity of cross-continental *P* recycling. Interior regions may be entirely dependent on the role ET plays in rainfall and water supply. In this sense, forest ecosystems represent *global public goods* that must find explicit expression in public policy.

Anthropogenic atmospheric climate change renders these relationships even more poignant. For the policy-maker, climate change poses significant challenges. As illustrated before, forest–water interactions have been poorly understood and the climate change mitigation and adaptation benefits of forest–water interactions remain poorly integrated into adaptation-related policy frameworks (Ellison, 2010). The few forest–water interactions successfully integrated into policy efforts are typically less controversial (e.g., riparian forest buffer zones mitigating nutrient and sediment runoff to streams and lakes). Other forest–water interactions remain a matter of significant debate. This is true not only for forest cover and water yield, but for a number of additional forest–water interactions such as the net effect of forest cooling and albedo warming, rainfall infiltration and groundwater recharge.

Recognizing that green water flows belong to the supply-side in forest hydrology models has important implications for current policy initiatives and understanding the impact of different land-use traditions and land conversions. In particular, we draw brief attention to current discussions of the development of measurement techniques to determine product-based water footprints (Hoekstra *et al*., 2009), the valuation of ecosystem services in the context of their potential role in water-pricing strategies, as well as the development and use of forestry as a tool for climate change mitigation and adaptation.

The relationship of forest cover to water yield is not well represented in a number of contemporary efforts to address issues related to concepts of ‘virtual water’ and the ‘water footprint’ (Allan, 1998; Merret *et al*., 2003; Hoekstra *et al*., 2009). The *Water Footprint Manual* (Hoekstra *et al*., 2009), for example, regards ET as a cumulative cost over the lifetime of given products (biomass, paper, food crops) rather than as a contribution to the atmospheric moisture vapor budget. While the impact of land conversions (in particular from forest to cropland) should be considered in terms of their impact on the evaporation potential per unit of land, forest-based products should either be thought of as comparatively neutral or even as providing significant contributions to the global water budget. The *ET-multiplier*, in particular, provides at least a preliminary estimate of its potential impact. The evaluation of ecosystem services confronts similar problems. Including ET on the *demand*- or *cost*-side of the water budget equation rather than on the supply-side has a significant (negative) impact on the overall valuation of ecosystem services.

Water-pricing strategies are essentially intended to help rationalize water use and help recover the costs of its production (OECD, 2010). In the context of the EU WFD (2000/60/EC), water-pricing strategies are intended to assist in the more rational use of water resources, in particular where EU member states are confronted with water scarcity problems. Although the approach represents an important step forward, the concept and approach of *cost recovery* for water provision is new in Europe (and many other countries) and there are significant implementation problems (Unnerstall, 2007). As Unnerstall (2007) notes, the recovery of *environmental* and *resource* costs is based entirely on *negative* impacts to the environment and not on the concept of the beneficial role of ecosystem services. No mechanism ensures that ecosystem services be rewarded and materially supported in water-pricing strategies. As such, it is also not likely that water-pricing revenues will flow back to and thus support ecosystem preservation and/or creation (see also Ellison, 2010).

The role of forests and forest cover is likewise disconnected from current discussions of drought management strategies in the EU and elsewhere. Dai (2010), for example, neglects potential connections between forest cover and drought potential. Presumably one reason is that traditional measures of drought vulnerability place forest ecosystems on the *demand-side*: ET represents a drain on the water budget. And, as Dai notes, one of the *presumed* strengths of the Palmer Drought Severity Index (PDSI), intended to measure departures from normal moisture conditions, is its recognition of the ‘demand’ impact of ET. This approach fails to recognize supply-side factors. Likewise, European Commission recommendations of potential drought management strategies and their inclusion in the planning aspects of the EU WFD fail to discuss forests from either the demand- or the supply-side.

Finally, although some countries have tapped large sums from the EAFRD (the European Agricultural Fund for Regional Development) to fund significant afforestation programs – in particular Spain, where total forest cover has increased by some 60% – the principal focus of afforestation strategies has been either on the carbon sequestration potential of forests or their value as sources of bioenergy. Based on our findings, the potential use of forests as an adaptation tool, in particular for promoting increased precipitation and combating drought, has been greatly underemphasized.

The recent EU Green Paper *On Forest Protection and Information in the EU: Preparing Forests for Climate Change* suggests a potential shift in the perception of the benefits of forest–water interactions, ‘Forests … play a major role in the atmospheric circulation and the water cycle on land and may have a role in mitigating regional climate, desertification and water security problems’ (European Commission, 2010, p. 10). However, as suggested by the range of policy discussions above, as well as European Environment Agency discussions of water scarcity (see, e.g., EEA, 2009), this perception is anything but deeply rooted.

## Conclusions

While a general perception in the current literature seems to be that forests reduce water availability, this conclusion is not well supported by the evidence. Placed in a larger regional and global context, forest–water interactions play a pivotal role in supplying the atmospheric moisture that becomes precipitation in the hydrologic cycle. Forests, wetlands and the ET they produce are one of the principal drivers of *P*. Without forests and wetlands, *P* will be significantly diminished. OE is not sufficient to provide adequate moisture vapor for all terrestrial regions. In particular, summertime *P* in many regions is predominantly driven by the ET regime.

As current and potential forest cover influence global *P* and the intensity of the hydrologic cycle, forests must be thought of as *global public goods*. Moreover, as the total amount of available ET (and thus *P*) can be influenced through ecosystem preservation/creation, strategies designed either to achieve this goal or remove existing forest cover (reforestation, afforestation and deforestation) have *transboundary* implications for local and global ET and the water regime. Thinking about ‘ecosystem services’ only in terms of local *water consumption* or the *demand-side* and not in terms of the large-scale *creation of water supply* explicitly limits our understanding of environmental processes and the central importance of ecosystems more generally. The *demand-side* of the water budget, however, remains important at the local scale and strategies for improving the efficient exploitation of both green and blue water resources must be considered. However, the most important *ecosystem service* is presumably the production of ET and the cross-continental transport of moisture vapor. These raise *P* and promote greater water availability, thus directly impacting potential blue water consumption.

Changing land use patterns, both in the developing and in the developed world, should be subject to increased scrutiny. Progressive deforestation and wetland destruction have direct implications for the global hydrologic cycle. In this regard, maintaining and/or significantly increasing current levels of forest cover seems advisable. Deforestation, due to its impact on the ET regime, on soil degradation and loss, and reduced soil water retention represents a significant threat to planetary survival. Increasing forest and wetland cover is likely to have beneficial feedbacks on regional water budgets. Thus, along with accepted forest ecosystem functions (such as carbon sequestration, climate mitigation, biodiversity preservation and fossil fuel substitution through bioenergy), forests play an important role in helping manage the world's water regime.

These are issues of great moment. Appropriate valuations of ecosystem services or calculations of the water footprint of forests and forest-based products can have significant impacts on future forest use. Water-pricing strategies being developed in the context of the EU WFD should consider the impact of forests on water supply and propose relevant guidelines that accurately reflect the impact of ecosystem services. Knowledge gaps and inadequate conceptual models can create difficulties in accurately identifying forest–water interactions and have important policy-relevant consequences. Inadequate or faulty understandings of the impact of forest cover on water yield can have long-term impacts on the cost and structure of policies governing water use, forest growth and the spread (or loss) of ecosystems. Global warming further intensifies the salience of issues related to forest ecosystem services and water supply.
